# Bolstering emergency response capacities in Africa: AVoHC-SURGE responders in action

**DOI:** 10.11604/pamj.supp.2025.50.1.47573

**Published:** 2025-04-23

**Authors:** Fiona Braka, Ishata Conteh, Radjabu Bigirimana, Joseph Okeibunor, Abdu Salam Gueye

**Affiliations:** 1 World Health Organization, Regional Office for Africa, Emergency Preparedness and Response Programme, Brazzaville, Congo,; 2 Africa Centres for Disease Control and Prevention, Addis Ababa, Ethiopia

**Keywords:** Mpox, Marburg, response, public health emergencies

The African Region faces increasing risks attributable to emerging and re-emerging public health threats. Consequently, the region has accelerated processes in strengthening emergency response capacities since 2022, building on investments made during the COVID-19 pandemic. The African Health Volunteers Corps (AVoHC) and Strengthening and Utilizing Response Groups for Emergencies (SURGE) initiative is one such process. It addresses major gaps in response, including the health emergency workforce, logistics, supplies, coordination, and community engagement. To date, 2,321 national multi-disciplinary responders (AVOHC-SURGE) have been enrolled across 29 countries. Between June 2022 and December 2024, AVoHC-SURGE responders responded to public health emergencies in 17 countries and 4 in 13 African countries.

## Commentary

As the world grapples with emerging and more frequent public health threats, the African Region continues to carry the larger brunt. The inherent risks and variability in health systems to absorb the shocks of disease outbreaks, adverse climate-related consequences, and forced displacements pose greater vulnerabilities for the region. Over the past three years, the worst drought in over four decades was recorded in the Horn of Africa, cholera outbreaks surged at a massive scale in southern Africa, and unprecedented flooding was witnessed in West and Central Africa, causing substantial health and socio-economic consequences [1,2]. These realities underscore the growing urgency for countries to build their resilience to mitigate the consequences of health emergencies.

### Regional approach driven by national governments

Since 2022, countries in the African Region have been accelerating efforts to strengthen capacities for emergency response, building on the investments made during the COVID-19 pandemic. A Regional Strategy for Health Security and Emergencies 2022-2030 was endorsed by African Ministers of Health in 2022 [3]. Underpinning the Regional Strategy are initiatives aimed at strengthening preparedness, detection, and response to emergencies. The African Health Volunteers Corps (AVoHC) and Strengthening and Utilizing Response Groups for Emergencies (SURGE) flagship initiative addresses major gaps hindering effective response: health emergency workforce, logistics and supplies, coordination, and community engagement [4].

Through a consultative and inclusive process with governments and stakeholders, WHO AFRO has supported 30 countries in the African Region, to date, to contextualize the SURGE initiative to address national capacity gaps for effective response. The aim is to identify and equip at least 3000 emergency responders in the region by 2026, ready to deploy within 24-48 hours of notification of an emergency. In the instance that country capacities are overwhelmed or need more specialized expertise not available locally, the initiative has established a low-cost mechanism for deployment of responders across borders through the Global Outbreak and Response Network (GOARN) [5]. To date, 2,321 national multi-disciplinary responders (AVOHC-SURGE) have been enrolled across 29 countries. Seventy-five percent (75%) of the responders are from Ministries of Health, while 25% are from other sectors. Women are increasingly involved in emergency response; however, a large disparity still exists overall, with only 30% of the AVoHC-SURGE team currently female. The multisectoral involvement has ensured that all aspects of the emergencies are addressed, fostering a comprehensive and sustainable response that not only tackles immediate health response needs but also supports long-term recovery and resilience.

### AVoHC-SURGE responders in action

**Figure 1:**
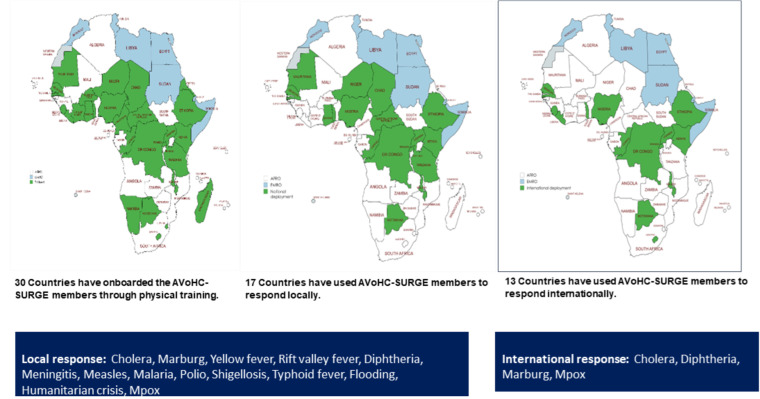
public health emergencies in the African Region between June 2022 to December 2024

Between June 2022 - December 2024, AVoHC-SURGE responders responded to 14 public health emergencies in 17 countries locally and 4 public health emergencies in 13 countries within the African Region (Figure 1). Emergencies ranged from disease outbreaks to humanitarian crises. The rapid deployment of emergency responders has contributed to more effective responses and a reduction in time to respond, ultimately saving lives. For instance, in September 2022, a cholera outbreak in Niger showcased the rapid deployment capabilities of the trained AVoHC-SURGE responders. A response team, equipped with the necessary supplies, was deployed within 48 hours of the first confirmed case. This rapid action confined the outbreak to a few districts in Maradi and Zinder regions, with 70 cases and two deaths recorded over six weeks - a stark difference from a previous cholera outbreak in March 2021 that spread to seven (7) regions with over 3,000 cases and 150 deaths over 12 months [6,7]. Additionally, Mpox-affected countries (CAR, Uganda, Rwanda, DRC, Ghana, Kenya) with trained AVoHC-SURGE responders have utilized them to support the response. On the other hand, WHO AFRO has also deployed 9 AVoHC-SURGE members from Congo, Senegal, and Togo to support the Mpox response in the DRC. Thirteen (13) countries (Botswana, Ethiopia, DRC, Kenya, Senegal, Togo, Congo, Nigeria, Rwanda, Malawi, Sierra Leone, Liberia, and Uganda) in the region have deployed their responders to support other countries.

During the recent Marburg outbreak in Rwanda in 2024, 39 national responders were deployed from Liberia (9), Uganda (11), and Sierra Leone (19). With the support and cooperation of the Governments of Rwanda, Liberia, Uganda, and Sierra Leone, WHO facilitated the rapid deployment and integration of the team to support critical pillars of the response. The Marburg outbreak was successfully contained within 11 weeks. This regional deployment approach has not only contributed to augmenting the ongoing response workforce capacity of DRC and Rwanda, amongst others, but also demonstrated the spirit of solidarity amongst countries during critical times.

### Future perspectives

The increasing risk of public health emergencies, exacerbated by urbanization, economic integration, environmental degradation, climate change, natural disasters, and conflict, necessitates urgent action to build local capacities for effective response and resilience across all countries. The SURGE initiative has provided not only a platform to bolster response capacities in Africa but has also positioned countries to address the diminishing resource envelope by promoting the use of local capacities and utilizing low-cost modalities for deployment, such as the GOARN. However, further efforts are required to strengthen capacities at the sub-national level to complement national-level systems. Mechanisms for the rapid activation and deployment of responders also need fine-tuning to ensure efficiency. Sustained solidarity and collaboration across countries remain essential for enabling rapid detection and response. Building on the SURGE initiative achievements, countries can strive towards a more secure and resilient African region and world.

## References

[1] World Health Organization Global health risks: mortality and burden of disease attributable to selected major risks.

[2] United Nations Office for the Coordination of Humanitarian Affairs (OCHA) West and Central Africa: Flooding Situation Overview - as of 5 October 2024.

[3] World Health Organization (2022). Regional strategy for health security and emergencies 2022-2030: report of the Secretariat. Regional Office for Africa.

[4] Conteh IN, Braka F, Assefa EZ, Daniel EO, Ngofa RO, Okeibunor JC (2024). Strengthening and utilizing response groups for emergencies flagship: a narrative review of the roll out process and lessons from the first year of implementation. Front Public Health.

[5] Gueye AS, Okeibunor J, Ngofa R, Conteh I, Onyeneho N, Mbainodji N (2024). Willingness of WHO staff to work in health emergencies in the African Region: opportunity for phased deployment of staff and ensure continuity of health services. Pan Afr Med J.

[6] World Health Organization First deployment of the SURGE team in Niger in response to the cholera epidemic.

[7] United Nations Trade & Development Economic Development in Africa Report 2018.

